# Githinji Gitahi: developing resilient health systems for universal coverage

**DOI:** 10.2471/BLT.24.030724

**Published:** 2024-07-01

## Abstract

Githinji Gitahi talks to Gary Humphreys about the value of cross-sectoral collaboration and health system assessment in the drive towards universal health coverage (UHC).

*Q: You grew up in a small rural village in Kenya, the child of farmers. How did that upbringing inform your world view and career choices?*

A: It made me acutely aware of the challenges faced by certain communities in accessing quality health care. And I would say that a desire to make a difference in the lives of people, particularly those who find themselves marginalized or excluded, has guided me in most of the projects I have worked on – certainly the work I do with Amref.

*Q: What is Amref’s core mission?*

A: To catalyse and drive community-led and people-centred primary health-care systems development, while also addressing the social determinants of health. We are the largest Africa-based international health development organization, and deliver health services and training to over 30 million people across the continent annually. Tackling access issues is reflected in all our work going right back to 1957 when the Flying Doctors of East Africa first brought health services to remote communities.

*Q: You have held positions of responsibility across companies and organizations in quite different sectors. How has that experience informed the work you do with Amref, particularly regarding UHC?*

A: Well, I started out as a clinician, but I very quickly became interested in what was causing the diseases and conditions of my patients. That led me to look outside the walls of the clinic to explore what have come to be known as the social determinants of health. I’d go out into the community and into people’s workplaces to try to understand the risks they faced. Early on in my career I also became very interested in how we, as a hospital, were meeting the needs of the communities we served. That steered me towards management roles, and eventually into hospital administration. I also worked in the health insurance arm of a health service provider, which gave me insights into the financing aspects of health service provision and got me thinking about how to make sure that essential health services were affordable to people paying low premiums.

“You can only assess what you can see.”

*Q: You later worked with Glaxo SmithKline (GSK) in marketing and product management roles. What lessons did you learn there?*

A: Principally, I learned about essential medicines from the manufacturer’s side of the fence and developed an understanding of the marketplace. However, I also worked on access there and, during my tenure, the company developed a high-volume low-margin pricing policy. Between 2001 and 2007 there were price reductions of up to 70% in some products, including essential medicines like antibiotics.

But I’d like to stress the value of multisectoral experience in developing collaborative approaches to tackling public health challenges. A lot of the work we do at Amref involves bringing people together, often people from different disciplines with different missions and imperatives. Having worked in different roles in different sectors I think helps me see the opportunities for collaboration and has also given me the tools to enable it. At GSK, for example, I worked on bridging research and clinical practice for better patient outcomes and served as a liaison between scientists, regulators and consumers. At Smile Train International, I focused on developing partnerships and programmes to provide corrective surgery for children with cleft lips and palates. One of the main challenges we faced with Smile Train was over-reliance on short-term, donor-driven medical missions. To address that issue, we focused on partnering with local hospitals and medical institutions to facilitate the training of local surgeons, building local capacity in countries such as the Democratic Republic of the Congo, Ethiopia, Ghana, Kenya, Nigeria and Uganda.

Lack of transportation was another big challenge. The annual surveys we did revealed that some patients had to travel up to1000 kilometres, and often required overnight accommodation. That finding really brought home to me the fact that access challenges exist outside the physical health facilities and, as a result, our health system assessments started to be more holistic, incorporating indicators such as availability of transportation and even societal permissions, such as husbands allowing wives to bring their children for treatments.

*A: You mention health system assessment. What part does that play in your work at Amref?*

A: Health system assessment, in the sense of taking a view of the whole, is obviously key. Without it, there is a tendency to fall into the kind of silo thinking that has hampered health system development in the past. This is often attributed to approaches taken by vertical programmes, but I would say that the policy positions developed under the MDGs (millennium development goals) also played a role. As many of your readers will remember, the MDGs focused on just eight goals, including three specific health goals: reducing child mortality, improving maternal health and combating HIV, malaria, and other diseases. When I joined Amref in 2015, the organization had a very vertical strategy in alignment with those goals. With the transition from the MDGs to the SDGs (sustainable development goals) it has become vital to consider the big picture and the connections between elements previously considered distinct, such as nutrition and climate change, to take just one example. Seeing the need for a shift in thinking, I attended a course on strategic perspectives for non-profit management at Harvard which helped me develop a more holistic, integrated approach, aligning with the UHC agenda. I also read *Health, Wealth, and the Origins of Inequality *by Angus Deaton, a book which profoundly influenced my understanding of UHC and equity, not least because of its insights regarding the way better data and a deeper understanding of the determinants of health can help frame more effective health and economic policies. These insights were pivotal in shaping Amref’s new five-year strategy that is focused on strengthening community health systems and increasing access to primary health care, but also prioritizes improving the livelihoods of women and young people and improving the social and structural conditions that impact health outcomes.

*Q: How does Amref approach health system assessment?*

A: We have developed our own approach, drawing on different methods and resources, including the World Health Organization's Service Access and Coverage (SAC) index, which aims to provide a clear, quantifiable measure of how effectively health services are being delivered and accessed by populations. However, it is important to point out that you can only assess what you can see – what you have data on. In Kenya, the data gathered by community health workers and posted on what we refer to as Community Health Blackboards at health facilities have been extremely helpful, highlighting gaps in vaccination, antenatal care and affordability. Data compiled by the Ministry of Health have also been useful, revealing for example, that only one person in five in Kenya had health insurance in 2017.

*Q: To what extent do the countries that Amref works with make use of health system assessment?*

A: Significant assessment data and evidence are available country by country – such as the United Republic of Tanzania’s data on facility financial autonomy, which revealed that facilities lacking financial autonomy struggle to provide quality care; or the data collected by Rwanda through its Imihigo initiative. However, challenges remain with regard to overall health system assessment in most countries.

*Q: Did the COVID-19 pandemic raise awareness of the need to assess capacity gaps and shortfalls in service provision?*

A: COVID-19 highlighted the shortcomings of the health systems, particularly in regard to vaccine distribution and oxygen provision. However, although there was a brief response to the situation, including an attempt to capture and compile information, things have since returned to ‘normal’, which is to say to underfunded systems which fail to meet the real needs of people, many of whom work in the informal sector.

*Q: What needs to change to address these issues?*

A: Several things, but one is the perennial issue of underfunding. In my role as co-chair of the UHC 2030 Steering Committee, I’ve witnessed the global push for countries to move towards UHC and it could not have been stronger. However, despite the progress in some countries, the drive towards UHC continues to be hampered by the limited resources made available. In Africa, for example, per capita expenditure on health reaches, at best, 50 United States dollars per capita, with many countries below that. This is in stark contrast with the thousands of dollars per capita spent in high-income countries. So there is clearly a need for greater resource mobilization but also a need to maximize the impact of the resources we have, starting with the provision of a limited range of services for the most vulnerable, providing a broader range of services as more resources become available. At Amref, we are assisting governments in learning how to make the most of their limited resources by purchasing health services strategically, to get more health for the money in the absence of more money for health. Rwanda has been particularly proactive in this area for some time, identifying the very poor and subsidizing them, integrating funds from the Global Fund into their health insurance plans, and maintaining a unified health financing strategy. Ethiopia, despite struggles with its community-based insurance system, has shifted focus to reducing disease severity by deploying community health extension workers, and establishing health posts to serve more people before their conditions worsen. Similar initiatives are seen in Malawi and Kenya, where there are efforts to support health system assistance through health insurance subsidies.

*Q: How optimistic are you that the UHC 2030 targets will be met in the countries in which Amref works?*

A: As assessed in the recent *WHO Results Report* for 2023, the world is off-track to meet the target of 1 billion more people benefiting from universal health coverage by 2025 and to meet the related sustainable development goals by 2030. The countries Amref works with are likely to be a part of that trend. Moving the needle on UHC requires leadership but it also requires governance structures that transcend electoral cycles. Implementing coherent UHC policies requires decades, while electoral cycles typically run for four or five years, disrupting long-term implementation and support. While Amref will continue to support its members in the certainty of making gains in certain areas, unless this short-term planning changes, they will continue to struggle.

